# Melatonin promotes hair regeneration by modulating the Wnt/β‐catenin signalling pathway

**DOI:** 10.1111/cpr.13656

**Published:** 2024-05-21

**Authors:** Yi‐Lin Niu, Yu‐Kang Li, Chen‐Xi Gao, Wen‐Wen Li, Li Li, Han Wang, Wei Shen, Wei Ge

**Affiliations:** ^1^ College of Life Sciences, Key Laboratory of Animal Reproduction and Biotechnology in Universities of Shandong Qingdao Agricultural University Qingdao China

## Abstract

Melatonin (MLT) is a circadian hormone that reportedly influences the development and cyclic growth of secondary hair follicles; however, the mechanism of regulation remains unknown. Here, we systematically investigated the role of MLT in hair regeneration using a hair depilation mouse model. We found that MLT supplementation significantly promoted hair regeneration in the hair depilation mouse model, whereas supplementation of MLT receptor antagonist luzindole significantly suppressed hair regeneration. By analysing gene expression dynamics between the MLT group and luzindole‐treated groups, we revealed that MLT supplementation significantly up‐regulated Wnt/β‐catenin signalling pathway‐related genes. In‐depth analysis of the expression of key molecules in the Wnt/β‐catenin signalling pathway revealed that MLT up‐regulated the Wnt/β‐catenin signalling pathway in dermal papillae (DP), whereas these effects were facilitated through mediating Wnt ligand expression levels in the hair follicle stem cells (HFSCs). Using a DP‐HFSCs co‐culture system, we verified that MLT activated Wnt/β‐catenin signalling in DPs when co‐cultured with HFSCs, whereas supplementation of DP cells with MLT alone failed to activate Wnt/β‐catenin signalling. In summary, our work identified a critical role for MLT in promoting hair regeneration and will have potential implications for future hair loss treatment in humans.

## Graphical abstract

Melatonin up‐regulates the release of Wnt3a and Wnt5a in HFSCs and down‐regulates the expression of SFRP2, which promotes the activation of the Wnt/β‐catenin signalling pathway in DP. The addition of melatonin significantly activates the Wnt signalling pathway in DP, which ultimately affects hair regeneration.

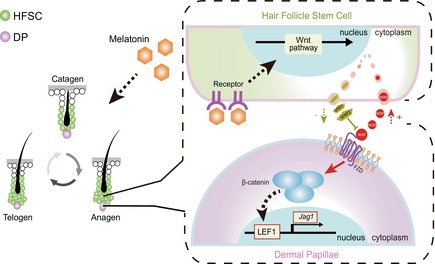

## 1 INTRODUCTION

The hair follicle (HF) is considered a miniature organ formed by neuroectoderm–mesoderm interactions,^1^, ^2^ and hair growth is primarily determined by transitions within a characteristic HF cycle consisting of three phases: anagen, catagen and telogen.^3^ During the cycle, intercellular communication between different cell types within the hair follicle, for example, dermal papillae (DP) cells, dermal sheath cells, hair follicle stem cells (HFSCs) and hair germ cells regulates the equilibrium of hair growth and hair loss.^4^, ^5^ Moreover, intercellular interactions are predominantly facilitated through signalling cascades, with several signalling pathways contributing to the regulation of hair follicle morphogenesis, including: Shh signalling, involved in hair follicle morphogenesis and late differentiation^6^, ^7^, ^8^; Notch signalling, involved in the fate determination of HFSCs^9^; BMP signalling, known to participate in the process of cellular differentiation^8^ and the Wnt signalling pathway, known as the master regulator of hair follicle morphogenesis.^10^, ^11^ Notably, Wnt signalling is activated when a Wnt ligand binds to a receptor/co‐receptor,^12^ resulting in the accumulation of β‐catenin in the cytoplasm,^2^ thereby activating the Wnt signalling pathway and regulating proteins and genes related to HF growth and development.^13^ Abrogation of Wnt signalling by removal of either *Tcf4* or β‐catenin, or by overexpression of the Wnt inhibitor Dickkopf‐1 results in the failure of hair follicle morphogenesis,^14^, ^15^, ^16^ thereby underlining the critical role of the Wnt signalling pathway during the hair growth cycle.

Melatonin (MLT) is a circadian pineal hormone that is also synthesized in the retina, skin, bone marrow and gut^17^; it acts as a key neuroendocrine regulator that couples coat phenotype and function to photoperiod‐dependent environmental and reproductive changes.^18^ Although MLT has been considered a sleep‐regulating hormone for several decades, recent studies indicate its involvement in many bodily functions including regulating hair growth^18^, ^19^; this is most evident in species that regulate hair growth according to seasonal changes in photoperiod. Continuous MLT treatment induces the transformation of hair follicles from the telogen to the anagen phase in New Zealand goats^20^; it also stimulates DNA synthesis in epidermal keratinocytes in hair follicle organoid cultures.^21^ Although there is suggestive *in vivo* evidence that MLT is an important regulator of hair growth, circulation, moulting and pigmentation in seasonal mammalian species, the way in which MLT regulates the hair growth cycle remains largely unknown.

In view of this, we designed an experiment to investigate the effect of MLT on hair regeneration in mice, by comparing the effects of different treatments on hair growth, and then tapping into the underlying molecular mechanisms. Our results showed that MLT can accelerate hair regeneration by regulating the Wnt signalling pathway, which promotes the transition of hair follicles from telogen to anagen.

## 2 MATERIALS AND METHODS

### 2.1 Animals

ICR mice were selected for this study and were purchased from Beijing Vital River Laboratory Animal Technology Co., Ltd. All mice were maintained in a specific‐pathogen‐free (SPF) animal room with 12 h light/12 h dark cycles, with free access to food and water before the experiments. All experimental procedures involved in this study were approved by the Animal Ethics Committee of Qingdao Agricultural University (No. SYXK‐2022‐0021).

### 2.2 Experimental design and mouse handling

Eight‐week‐old ICR male mice were used for the experiments. Firstly, the hair on the back of the mouse was shaved; secondly, the remaining stubble was removed with a depilatory cream to expose the bare skin of the back. MLT was administered to the relevant groups of mice via a corn oil gavage at 9:00 AM and the MLT receptor inhibitor, luzindole, via intraperitoneal injection at 3:00 PM every day for 20 consecutive days; the body weight changes of mice were evaluated during this period (Figure S1). The control group (CON) received a gavage of corn oil (Xiwang Group Company Limited, Shandong, China) and an intraperitoneal injection of saline. The MLT group, received a MLT dose of 40 mg/kg/day (Sangon Biotech, Shanghai, China) and a saline injection. The MLT receptor inhibitor (Inhib) group, received a corn oil gavage followed by the MLT receptor inhibitor luzindole (0.5 mg/kg/day, Sigma, MO, USA) intraperitoneal injection. The negative control group (MLT + Inhib) received the corn oil/MLT mixture (40 mg/kg/day) gavage and an intraperitoneal injection of luzindole (0.5 mg/kg/day). The samples were collected after 20 days of treatment.

### 2.3 RNA‐sequencing and data analysis

Samples collected 20 days after treatment were immediately snap frozen in dry ice for RNA‐sequencing (RNA‐seq). The Hiseq4000 platform was used for RNA‐seq. Quality control of raw data was analysed using FastQC to filter out low‐quality sequences to obtain a clean data set. Then the data were analysed using STAR software (v2.6.1b, NY, USA) to index the *Mus* reference genome and produce mapped reads (Table S1). Subsequently, the DESeq2 package was used to obtain differentially expressed genes (DEGs) between groups. Kyoto Encyclopedia of Genes and Genomes (KEGG) pathways and Gene Ontology (GO) terms with a *p*‐value <0.05 were taken to be significantly enriched.

### 2.4 Haematoxylin and eosin staining

The cut skin tissue blocks were first washed with 1× PBS (Solarbio, Beijing, China) to remove impurities on the skin surface, then fixed overnight in 4% paraformaldehyde (Solarbio) at 4°C, dehydrated with graded alcohol, embedded in paraffin and then sectioned at 7–8 μm with a microtome (Leica, Wetzlar, Germany). Xylene was used to remove paraffin from the sections before staining, after which the tissues were rehydrated through graded alcohols. Tissues were then stained with haematoxylin and eosin (H&E) according to the manufacturer's instructions (Beyotime, Shanghai, China). Finally, the sections were pressed and sealed with neutral resin and photographed under a microscope (Olympus, Tokyo, Japan).

### 2.5 Immunofluorescence

Samples were processed, embedded and sectioned as described for H&E staining. For immunofluorescence staining analysis, sections were first deparaffinized in xylene solution for 30 min and then rehydrated through graded alcohols. Antigens were then extracted with 0.01 M sodium citrate buffer (pH = 6.0) at 96°C for 10 min. After cooling to room temperature (RT), the samples were blocked with TBS containing 3% BSA (Solarbio) and 10% goat serum (Boster, CA, USA) for 30 min at RT, and then incubated with the primary antibody (Table S2) diluted in blocking solution overnight. The next morning, the slides were returned to RT, then the corresponding secondary antibody (Table S3) was added and incubated at 37°C for 1.5 h. The nuclei were stained with DAPI, and the slides were mounted with an anti‐fade mounting medium.

For immunofluorescence, the collected HFSC clones were first digested into single cells with trypsin. Subsequently, the obtained cells were fixed with 4% paraformaldehyde solution at RT for 30 min. Then, the single‐cell pellets were spun onto a microscope slide, and the slides were dried at 37°C. Following three washes with 1× PBS solution for 5 min each, the slides were blocked with PBS solution containing 5% BSA and 0.5% Triton‐X‐100 (Solarbio) for 45 min. The slides were then incubated with corresponding primary antibodies (Table S2), which were diluted in a blocking solution at 4°C overnight. The next morning, corresponding secondary antibodies (Table S3) were added, and the slides were incubated at 37°C for 1.5 h. Nuclei were subsequently stained using DAPI. Finally, the slides were mounted with an anti‐fade mounting medium (Boster), and photographs were taken using a Nikon (BX51, Tokyo, Japan) microscope.

### 2.6 Western blot

The skin tissues were first washed three times with ice‐cold PBS to remove contaminants after dissection; they were then soaked in RIPA (Beyotime) for lysing, the supernatant was collected after centrifugation and a quarter volume of 5 × SDS‐PAGE Sample Loading Buffer (Beyotime) was added to the supernatant and boiled in water for 5 min to denature the protein. Protein samples were separated with 10%–12% gel. After SDS‐PAGE, proteins were transferred to a PVDF membrane (Merck Millipore, MA, USA), and the membrane was blocked with TBST containing 5% BSA (Solarbio) at RT for 2–3 h and then incubated with the primary antibody (Table S2) overnight at 4°C. After washing with TBST, the primary antibodies were incubated with the corresponding secondary antibodies (Table S3) for 1.5 h at RT. Bands were visualized with an ECL kit (Beyotime), and gel band quantification was analysed using ImageJ software.

### 2.7 Preparation and cultivation of HFSCs

Seven‐day‐old mice were killed by cervical dislocation and then soaked in alcohol for 5 min to remove any bacteria. The whiskers around the mouth were cut with scissors, and tweezers were used to pull out the hair follicles. Hair follicles were washed with 1× PBS twice, then digested with trypsin (Thermo Fisher Scientific, MA, USA) for 10 min; after blowing on them with a pipette for 5 min, the digestion was stopped. The samples were centrifuged at 1700 rpm for 3 min, the supernatant was discarded and the cells were resuspended in DMEM/F12 (Gibco, NY, USA); they were then filtered using a 40 μm cell strainer (Biologix, Shandong, China). Cells were subsequently re‐centrifuged, the supernatant discarded and the cells were seeded in HFSC medium (DMEM/F12; 1% B27, Gibco, NY, USA; 20 ng/mL EGF, R&D Systems, MN, USA; 40 ng/mL bFGF, PeproTech, NJ, USA; and 1% Penicillin–Streptomycin Solution, Solarbio) and cultured using a suspension culture plate (Sarstedt, Nümbrecht, Germany). As the culture time extended, HFSC clones were gradually formed, whereas other somatic cells adhered to the bottom of the culture plate; after two passages, purified HFSC clones were obtained for downstream analysis.

For downstream treatments with MLT and luzindole, after 12 days of culture, the cells were harvested and re‐digested with trypsin down to single cells; then they were divided into three groups. The CON group continued to be cultured with the previous medium, and the MLT group used the previous medium containing 10 nM MLT. The MLT + Inhib group was further cultured with a medium containing 10 nM MLT and 10 μM luzindole. The cells were cultivated for 5 days; during this process, photos were taken with a camera mounted on a microscope to record the growth status of cells every day; finally, the samples were collected for downstream analysis.

### 2.8 Acquisition and cultivation of DPs

After obtaining individual hair follicles (the method was consistent with the acquisition of HFSCs), the tip of a 1 mL syringe was used to cut off the entire DP area at the root of the hair follicle; they were then transferred to 1× PBS and washed twice. Before culture, they were randomly divided into two groups, The CON group was cultured in HFSC medium, and the MLT group was cultured in HFSC medium containing 10 nM MLT. During culture, 500 μL HFSC medium was added to each well of 24‐well plates (Nest, Wuxi, China), and 10–20 DP tissues were cultured in each well. Samples were collected after 5 days of culture. When DP tissues and HFSCs were co‐cultured, after the HFSCs had been cultured for 12 days, they were divided into two groups and added to the 24‐well plate. The DP tissues were then placed on a Transwell (Corning, NY, USA) device. Subsequently, a transwell containing DP tissues was placed into the wells for co‐culture for the different groups, and samples were collected after 5 days of culture.

### 2.9 Statistical analysis

For statistical analysis, we used SPSS software (IBM Co., v20.0.0, NY, USA) with a one‐way analysis of variance followed by LSD multiple comparison tests. Data were visualized using GraphPad Prism 8 (GraphPad Software, Inc., v8.0.2, CA, USA) software, and significance was determined when *p* < 0.05. Differences are denoted by a, b, c and d in descending order. Any significant difference between two data sets is denoted by a different letter; if there was no significant difference between two data sets, this is denoted by use of the same letter.

## 3 RESULTS

### 3.1 MLT promoted hair regeneration

**FIGURE 1:**
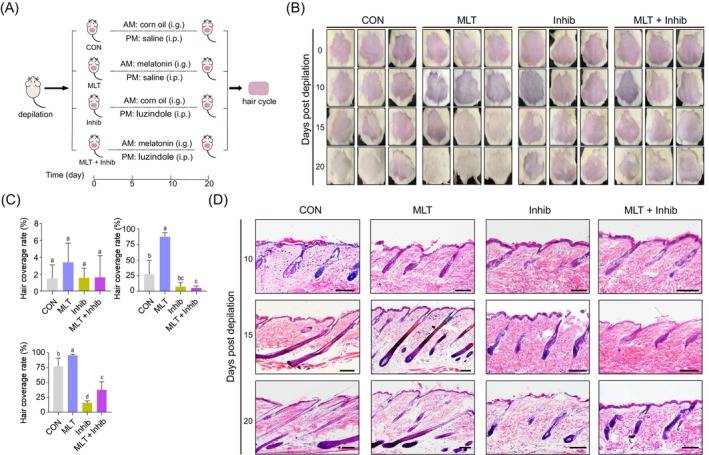
Preparation and handling of mouse models. (A) The mice were randomly divided into four groups after depilation, and the treatments were divided into morning and afternoon. The groups were handled as shown, continuously for 20 days. (B) Hair coverage of mice during treatment. Mice were imaged on days 0, 10, 15 and 20. (C) Quantification of hair coverage on days 10, 15 and 20. Hair growth on the backs of all individuals was recorded. For each group, at least five mice were included for statistical analysis; statistical significance was considered at *p* < 0.05. (D) Haematoxylin and eosin staining of different groups of skin at 10, 15 and 20 days after depilation. Scale bars, 100 μm. i.g., intragastrical administration; i.p., intraperitioneal injection.

To systematically investigate how MLT regulated hair growth, we first established a hair depilation mouse model using the methods previously described^22^ and investigated the effects of MLT and its MLT receptor antagonist luzindole. First, the back skin of 8‐week‐old ICR mice was shaved using a razor; then, mice were randomly divided into 4 groups (CON, MLT, Inhib and MLT + Inhib, with a minimum of 10 biological replicates in each group). For the different groups of mice, various treatments were administered over the next 20 days, divided into morning and afternoon sessions (Figure 1A). Briefly, for CON, mice were gavaged with corn oil (MLT vehicle) in the morning, followed by an intraperitoneal injection of saline in the afternoon. For MLT, mice received MLT (via gavage diluted into corn oil) in the morning followed by an intraperitoneal injection of saline in the afternoon. For Inhib, mice were gavaged with corn oil in the morning and followed by an intraperitoneal injection of MLT receptor antagonist luzindole^23^, ^24^ in the afternoon. For MLT + Inhib, mice were given MLT by gavage in the morning, followed by an intraperitoneal injection of MLT receptor antagonist luzindole in the afternoon. Daily recordings of the weights of the mice showed no significant differences among the four groups (Figure S1). Morphologically, after the 20 days experimental period (Figure 1B), the CON group had obvious new hair growth covering the surface of the skin, whereas for the MLT group, noticeable new hair coverage was observed at around 15 days. Meanwhile, at 20 days after depilation for mice in the luzindole‐treated groups (Inhib and MLT + Inhib groups), the formation of new hair on the skin surface was significantly reduced and was clearly less than that in the CON and MLT groups.

We further compared hair coverage in the skin tissues at different time points after depilation (Figure 1C), aiming to systematically evaluate the impact of MLT on hair follicle development. Notably, at 10 days after depilation, there were no significant differences regarding MLT or luzindole treatment, whereas on the 15th day after depilation, MLT‐treated animals showed a significantly higher hair coverage rate compared with the remaining groups. On the 20th day after depilation, it was apparent that both CON and MLT groups showed significantly higher hair coverage rates compared with luzindole‐treated groups (Figure 1C). Notably, the hair coverage rate in the MLT group was significantly higher than for CON, whereas in MLT + Inhib, the hair coverage rate was also significantly higher than in Inhib (Figure 1C). Together, these data suggest that MLT treatment promoted hair regeneration in the depilated mouse model and a promoting effect of MLT on hair follicles was achieved through MLT receptors, indicating their critical role in regulating hair regeneration.

To provide in‐depth insights into how MLT treatment affected hair growth (Figure 1D), we further performed tissue section analysis from different time points. On the 15th day after depilation, it was observed that hair follicles in the MLT and CON groups mainly developed to the anagen stage. There were also a greater number of hair follicles in the anagen phase in the MLT group when compared with CON. While for the groups supplemented with MLT receptor antagonist luzindole, the hair follicles were mainly in the telogen stage. On the 20th day after depilation, the hair follicles in the CON and MLT groups were mainly in the anagen stage and showed little difference. By comparison, for the Inhib and MLT + Inhib groups, the hair follicles were arrested in the telogen phase of the hair cycle, which was consistent with morphological observations of hair growth.

### 3.2 MLT promoted hair regeneration through modulating the hair cycle

**FIGURE 2:**
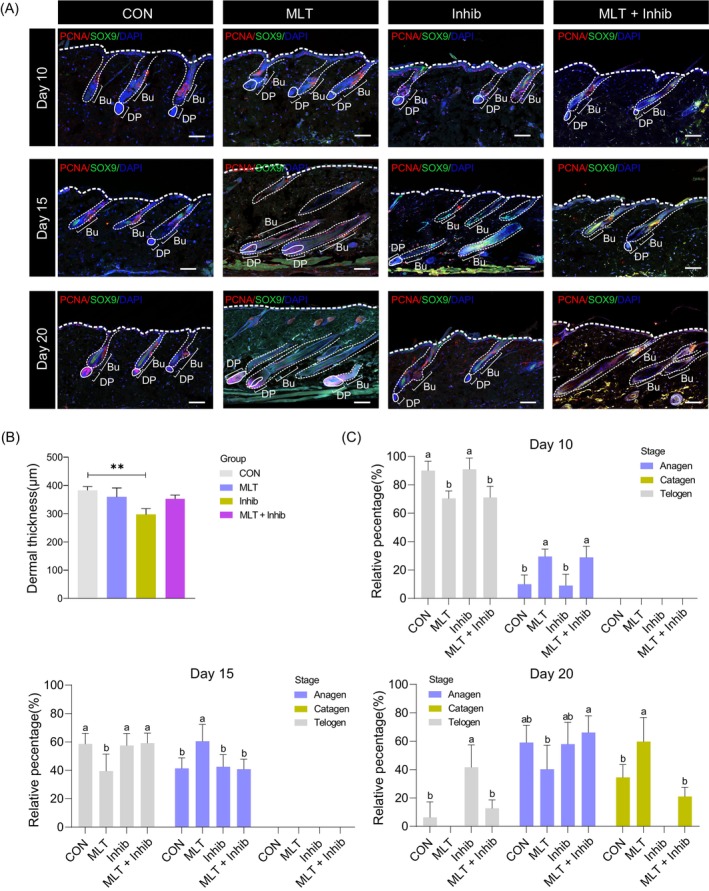
Immunofluorescence of the skin and statistics of the hair follicle stage. (A) Immunofluorescence co‐staining of PCNA and SOX9 on skin samples of different groups at 10, 15 and 20 days after depilation. Scale bars, 100 μm. (B) Statistics of dermal layer thickness, **p < 0.01. (C) Statistical analysis of hair follicle developmental stage in different groups at 10, 15 and 20 days after depilation. For each group, at least four mice were included for statistical analysis. Bu, bulge; DP, dermal papillae.

After determining that MLT promoted hair regeneration using the depilation mouse model, we next performed immunofluorescence analysis using the hair follicle stem cell marker SOX9^25^ and cell proliferation marker PCNA (Figure 2A)^26^, ^27^ to provide deeper insights into how MLT promotes hair regeneration. Notably, 10 days after depilation, the staining pattern of SOX9 and PCNA showed no difference between the various groups. Although on the 15th day after depilation, in the MLT group, there were more PCNA‐positive cells in the DP region compared with the CON and luzindole‐treated groups. For the luzindole‐treated groups, PCNA‐positive cells were significantly lower in number than in the CON and MLT groups. On the 20th day after depilation, PCNA‐positive cells in the MLT group were predominantly located in the DP region, whereas for the CON group, these cells were mainly located in the DP and bulge region. For the luzindole‐treated groups (Inhib and the MLT + Inhib groups) PCNA‐positive cells in the DP were decreased when compared with the CON and MLT groups. We further evaluated the expression level of cell proliferation marker Ki67 on the DP region of single hair follicles among various groups at 20 days after depilation (Figure S2A); the results were also consistent with our PCNA staining assay. Interestingly, when comparing skin thickness, there was no significant difference for the epidermis (Figure S2B), but in the Inhib group, the thickness of the dermis was less than in the remaining groups (Figure 2B). Together, these data suggest that MLT may regulate hair regeneration through DP cells.

Based on the immunofluorescence analysis, we further analysed the hair cycle between groups (Figure 2C). Notably, at 10 days after depilation, the hair follicles were mainly in the telogen stage, whereas at 15 days after depilation, more than half of the hair follicles in the MLT group had entered the anagen stage, with the percentage of anagen stage hair follicles in the other three groups remained below 50%. At 20 days after depilation, the percentage of anagen hair follicles in the MLT group was reduced and catagen stage hair follicles became dominant (greater than 50% of total hair follicles). Interestingly, for the Inhib group, the hair follicles were mainly at the anagen and telogen stages, indicating a delayed progression of hair cycling, whereas for the MLT + Inhib group, we observed that about 20% of hair follicles had entered the catagen stage, indicating that supplementation of MLT partially abrogated luzindole induced delaying of the hair cycle.

### 3.3 MLT promoted the hair cycle through mediating hair cycle‐related gene cascades

**FIGURE 3:**
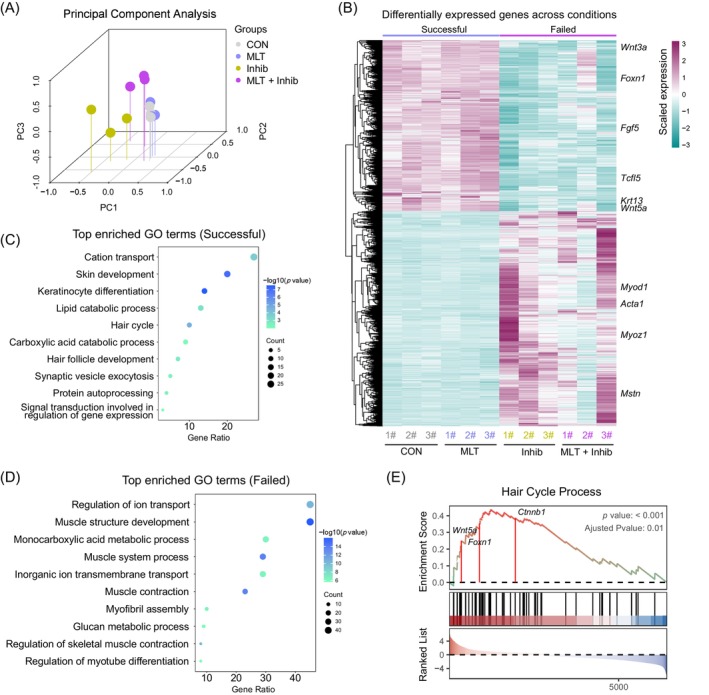
Transcriptome analysis of skin tissues from different groups. (A) The principal component analysis (PCA) plot of different groups at 20 days after depilation. (B) Heatmap showing the top 200 differentially expressed genes on day 20 skin between the Successful group and the Failed group. Control group (CON) and melatonin (MLT) groups were placed into the Successful group, while the MLT receptor inhibitor (Inhib) and negative control (MLT + Inhib) groups were assigned to the Failed group, based on hair growth status. (C) Gene Ontology (GO) enrichment results of up‐regulated genes in the Successful group. (D) GO enrichment results of up‐regulated genes in the Failed group. (E) The gene set enrichment analysis (GSEA) plot illustrating the enrichment of hair cycle process‐related genes between the Successful group and the Failed group.

To provide in‐depth insight regarding how MLT regulates the hair cycle, we next performed transcriptome analysis using skin tissues from various groups at 20 days after depilation. First, we performed principal component analysis (PCA) to provide a preliminary insight into how MLT treatment affected global gene expression profiles (Figure 3A). The results showed that PCA analysis successfully separated the four groups, thus indicating strong reproducibility among all samples analysed. Furthermore, it was observed that the Inhib group clustered closely with the MLT + Inhib group, indicating similar gene expression profiles between the Inhib and MLT + Inhib groups. Conversely, the MLT group clustered closely with samples from the CON group, suggesting similar gene expression profiles between the MLT and CON groups. This observation is also consistent with our morphological findings, which demonstrated that the CON and MLT groups displayed normal hair growth, whereas the blockade of MLT by luzindole compromised hair regeneration ability.

To further investigate how MLT promotes hair regeneration, we first classified the experimental mice into ‘Successful’ groups (including CON and MLT groups, displaying normal hair growth with endogenous and exogenously supplemented MLT) and ‘Failed’ groups (including Inhib and MLT + Inhib groups, exhibiting compromised hair growth with MLT receptor antagonist), based on the hair regeneration status. We then performed a differential gene expression analysis (Figure 3B), and after DEG analysis, distinct gene expression profiles between the Successful and Failed groups were observed. Specifically, the Successful group had significantly up‐regulated key genes influencing hair growth, for example, *Foxn1* and *Fgf5*.^28^, ^29^ Notably, we also observed up‐regulation of Wnt signalling ligands *Wnt3a* and *Wnt5a*. We further performed a gene functional enrichment analysis to unveil the biological processes and pathways associated with MLT‐mediated hair regeneration. GO enrichment analysis using group‐specific up‐regulated DEGs indicated that the CON and MLT groups showed a high percentage of shared DEGs and GO terms, while the Inhib group showed no shared DEG terms compared with the remaining groups (Figure S3A,B). For the MLT + Inhib group, we observed that, in comparison to the Inhib group, a partial overlap of genes with both the CON and MLT groups was apparent. Importantly, after DEG analysis between the Successful and Failed groups, gene functional enrichment analysis unveiled that the Successful group had significantly enriched genes involved in ‘Cation transport’, ‘Skin development’, ‘Hair cycle’ and ‘Hair follicle development’ (Figure 3C), whereas for the Failed group, genes enriched in GO terms of ‘Regulation of ion transport’, ‘Muscle structure development’, ‘Monocarboxylic acid metabolic process’ and ‘Muscle contraction’ were significantly up‐regulated (Figure 3D). Furthermore, gene set enrichment analysis also indicated that hair cycle process‐related pathways were significantly enriched in the Successful group. Notably, we found that key components of Wnt/β‐catenin signalling were significantly enriched in GSEA analysis (Figure 3E), including Wnt ligand *Wnt5a* and downstream effector β‐catenin, therefore indicating a critical role of Wnt/β‐catenin signalling during MLT mediated hair regeneration. Together, these analyses demonstrated that MLT plays an important role in regulating hair regeneration and promotes the hair cycle by upregulating key genes involved in hair cycle processes.

### 3.4 MLT activated Wnt/β‐catenin signalling in the hair follicle

**FIGURE 4:**
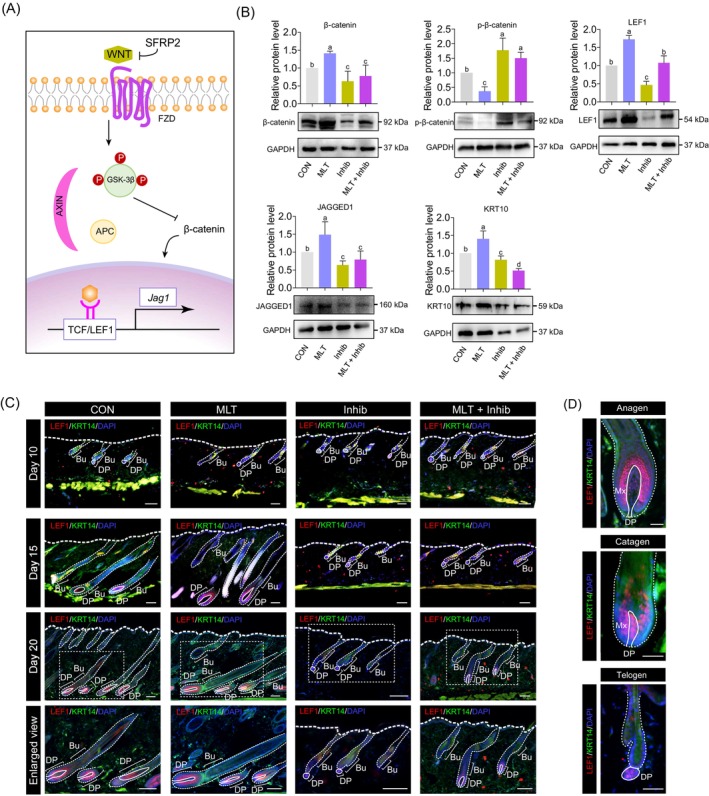
Wnt/β‐catenin signalling pathway regulates hair regeneration. (A) Schematic diagram illustrating how Wnt ligands activate the Wnt/β‐catenin signalling pathway. (B) Western blot showing β‐catenin, p‐β‐catenin, LEF1, JAGGED1 and keratin‐10 (KRT10) expression levels in skin tissues of different groups at 20 days after depilation. (C) Immunofluorescence co‐staining of LEF1 and cytokeratin‐14 (KRT14) in skin samples from different groups. The magnified view at the bottom corresponds to the area indicated by the white dashed rectangle on the 20th day after depilation. Scale bars, 100 μm. (D) Localization of LEF1 at different stages of the hair cycle; the solid lines indicate the dermal papillae (DP) region. Scale bars, 50 μm. FZD, frizzled receptor; APC, adenomatous polyposis coli; GSK‐3β, glycogen synthase kinase; Bu, bulge; Mx, matrix.

Wnt signalling plays a decisive role in the initial stage of hair growth.^10^, ^30^ β‐Catenin,^31^, ^32^, ^33^, ^34^ a key regulatory protein of the classic Wnt signalling pathway, regulates hair growth by activating the nuclear auxiliary transcription factor LEF1,^35^ and this activation further up‐regulates the downstream gene *Jag1* ^36^ in hair follicles (Figure 4A). Because transcriptome analysis revealed that Wnt/β‐catenin signalling‐related genes were significantly up‐regulated in the MLT group, we went on to investigate whether MLT promoted hair regeneration through mediating the Wnt/β‐catenin signalling in the hair follicle. To this end, we analysed protein expression levels of β‐catenin, p‐β‐catenin, LEF1 and JAGGED1 among different groups. Results showed that the expression level of β‐catenin, LEF1 and JAGGED1 was significantly up‐regulated in the MLT group (Figure 4B), whereas in the luzindole‐treated groups, these proteins showed the lowest expression level. In contrast, regarding the expression level of p‐β‐catenin, this was significantly up‐regulated in the luzindole‐treated groups. Together, these data demonstrate that the Wnt/β‐catenin signalling was significantly activated in the MLT group, whereas inhibition of MLT receptor by luzindole abrogated such effects. In addition, we examined the protein expression levels of keratin‐10 (KRT10), an early marker of cell differentiation in the basal layer of the epidermis.^37^ KRT10 expression level in the MLT group was significantly increased, whereas in the Inhib and MLT + Inhib groups, the expression level was significantly reduced. Together, these data suggest that Wnt/β‐catenin signalling was activated after MLT treatment in the skin, whereas treatment involving the MLT receptor antagonist luzindole significantly blocked Wnt/β‐catenin signalling.

Previous reports have demonstrated that Wnt/β‐catenin signalling plays an important role in skin homeostasis, and our results here also demonstrate that MLT activates Wnt/β‐catenin signalling during hair regeneration. However, the types of cells that are activated by MLT in the Wnt/β‐catenin signalling pathway remain unclear. To this end, we next performed immunofluorescence staining of the key Wnt/β‐catenin signalling player LEF1 and keratinocyte marker KRT14 among the various groups (Figure 4C). Notably, LEF1‐positive cells were mainly located at the DP region during early hair growth, as hair follicles entered the anagen phase; LEF1‐positive cells were also detected in the epithelial matrix (Mx), which is consistent with previous studies (Figure 4D).^38^ Interestingly, at 10 days after depilation, the staining pattern of LEF1 and KRT14 was similar among the groups. However, in the MLT groups, at 15 days after depilation, there were more LEF1‐positive cells compared with the CON and luzindole‐treated groups. For the Inhib and MLT + Inhib groups, the hair follicles were smaller than in the other two groups, and the number of LEF1‐positive cells was significantly lower. On the 20th day after depilation, the number of LEF1‐positive cells was similar between the CON and MLT groups. At this point, the LEF1 signal in the MLT + Inhib group was also activated in DPs but showed a weak staining pattern in the Inhib group. We further performed β‐catenin staining (Figure S4); the results also showed that β‐catenin‐positive cells were mainly located at DPs of the CON, MLT and MLT + Inhib groups, whereas for the Inhib group, β‐catenin‐positive cells were not observed around DPs. Together, these findings suggest that MLT promotes hair regeneration by activating Wnt/β‐catenin signalling in DPs.

### 3.5 Hair follicle stem cell‐derived Wnt ligands activated Wnt/β‐catenin signalling

**FIGURE 5:**
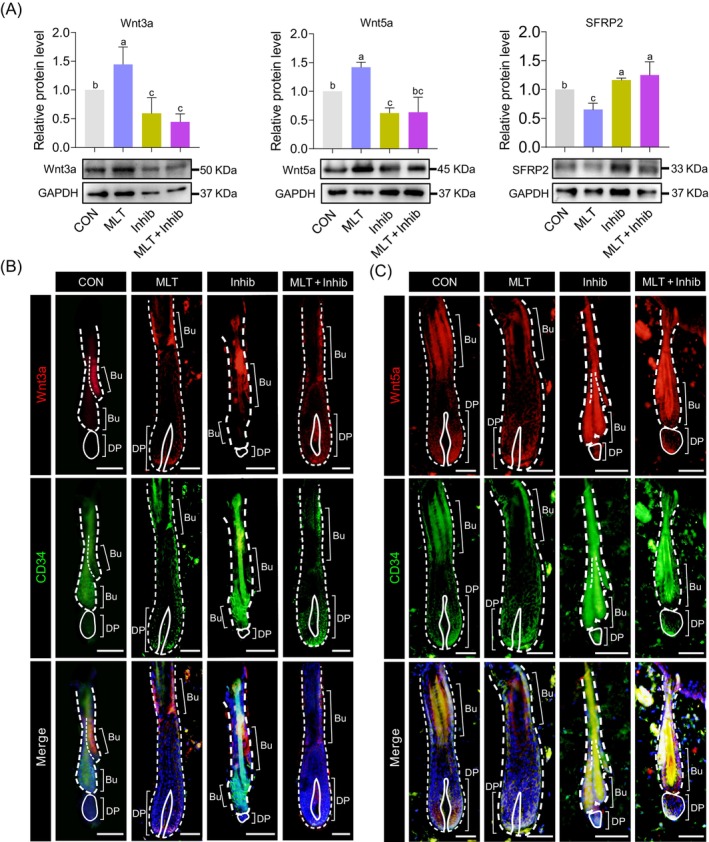
Expression levels and location of Wnt ligands. (A) The protein levels of Wnt3a, Wnt5a and SFRP2 in different groups at 20 days after depilation. (B) Immunofluorescence co‐staining of Wnt3a and CD34 of skin samples at 20 days after depilation. Scale bars, 50 μm. (C) Immunofluorescence co‐staining of Wnt5a and CD34 of skin samples at 20 days after depilation. Scale bars, 50 μm. Bu, bulge; DP, dermal papillae.

The Wnt/β‐catenin pathway is activated when a Wnt ligand binds to the corresponding receptor in a target cell; various ligands have been demonstrated to be involved in this process.^4^, ^39^ Published data shows that Wnt ligands activate the Wnt/β‐catenin signalling pathway in target cells through autocrine or paracrine mechanisms.^39^, ^40^, ^41^ Comparison of the gene expression levels of Wnt ligands between the Successful and Failed groups revealed that *Wnt3a*, *Wnt5a*, *Wnt11*, *Wnt4*, *Wnt6* and *Wnt9a* were all consistently up‐regulated in the Successful group when compared with the Failed group, with *Wnt3a*, *Wnt5a* and *Wnt11* significantly up‐regulated in the Successful group (Figure S5). To verify whether these Wnt ligands were involved during MLT‐induced activation of the Wnt/β‐catenin signalling, we further verified the expression levels of Wnt3a and Wnt5a at the protein level among various groups as these two ligands were involved in the activation of the Wnt signalling pathway.^42^, ^43^ Consistent with our transcriptome analysis, the results showed that these proteins were significantly up‐regulated in the MLT group (Figure 5A). Meanwhile in the luzindole‐treated groups, the expression level of these proteins was significantly down‐regulated when compared with the MLT group. We further evaluated the expression level of SFRP2, a well‐known Wnt antagonist that inhibits the binding of Wnt ligands to membrane receptors and thus inhibits the Wnt signalling pathway.^44^, ^45^ The results showed that SFRP2 was significantly up‐regulated in the luzindole‐treated groups. Together, these data suggest that MLT promotes hair regeneration through increased expression of Wnt ligands and that these ligands then activate Wnt/β‐catenin signalling.

To further explore which hair follicle cell types were direct targets of MLT‐induced Wnt ligand secretion, we co‐stained Wnt3a and Wnt5a with hair follicle stem cell marker CD34 using skin tissues at 20 days after depilation (Figure 5B,C).^46^, ^47^ The staining results unveiled a similar staining localization pattern between Wnt3a‐positive cells and Wnt5a‐positive cells, particularly in the CON and MLT groups, where these positive cells were predominantly located in the bulge region of the hair follicle. Furthermore, it was found that Wnt3a and Wnt5a‐positive cells were co‐located with the hair follicle stem cell marker CD34. When combined with previous results showing that both β‐catenin and LEF1 are enriched in the bulge and DP region of hair follicles in the MLT group, these results suggest that MLT may activate Wnt/β‐catenin signalling through mediating Wnt ligand expression in HFSCs.

### 3.6 MLT activated the Wnt/β‐catenin signalling pathway in DPs by promoting the release of Wnt ligands from HFSCs *in vitro*

**FIGURE 6:**
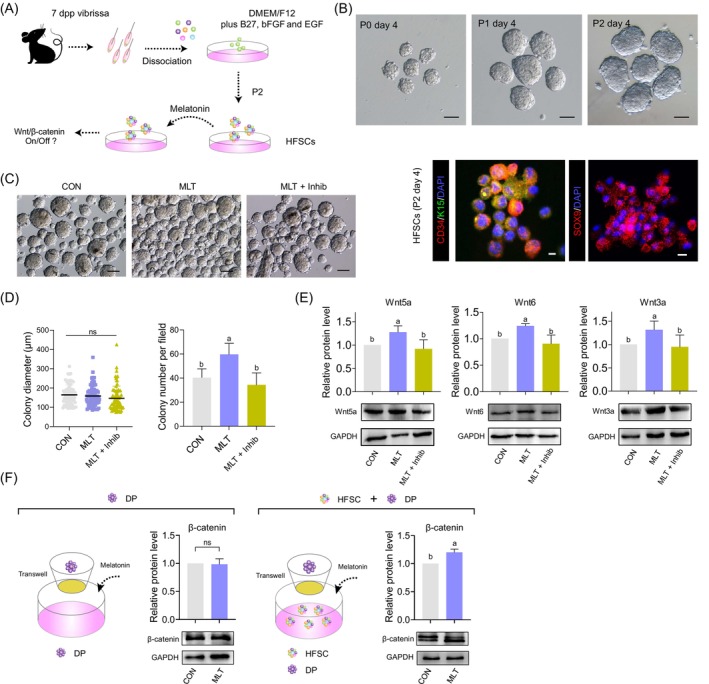
The impact of melatonin (MLT) on the interaction between hair follicle stem cells (HFSCs) and dermal papillae (DP). (A) Schematic diagram illustrating the major steps in hair follicle stem cells isolation and culture *in vitro*. (B) Top panel: The morphology of colonies formed by HFSCs cultured for 4 days at passages P0, P1 and P2. Scale bars, 100 μm. Bottom panel: Immunofluorescence staining of CD34, keratin‐15 (K15) and SOX9 of HFSCs cultured for 4 days at P2. Scale bars, 10 μm. (C) The morphology of colonies formed by HFSCs cultured for 4 days at passage P2 after MLT and luzindole exposure. Scale bars, 100 μm. (D) Statistical analysis of colony diameter and colony number per field after MLT and luzindole exposure. (E) Western blotting and quantification of Wnt5a, Wnt6 and Wnt3a levels in the HFSCs in different groups. (F) Western blot illustrating the effects of MLT on the expression level of β‐catenin in dermal papillae (DP) cells based on a DP‐HFSCs Transwell co‐culture system. dpp, days post partum.

To further verify whether MLT activated the Wnt/β‐catenin signalling pathway in DPs through HFSCs, we further tested this hypothesis using an *in vitro* culture model. First, we established an HFSC *in vitro* culture system as previously described (Figure 6A).^48^, ^49^ This provided a means to test whether MLT exposure promoted the up‐regulation of Wnt ligands. Specifically, HFSC culture *in vitro* showed representative round colonies, and with prolonged culture, these colonies increased in size (Figure 6B, top panel). Immunofluorescence staining analysis of canonical HFSC markers CD34, KRT15 and SOX9 also confirmed their HFSC identity (Figure 6B, bottom panel), which was consistent with a recent report.^25^, ^50^

Based on the HFSC *in vitro* culture system, we then questioned how MLT affected HFSCs. Firstly, we tested varying concentrations of MLT during *in vitro* culture to evaluate whether MLT exposure affected HFSCs (Figure S6A). After 5 days of *in vitro* culture, it was observed that 10 nM MLT exposure significantly increased HFSC colony diameter and the expression of HFSC marker CD34 (Figure S6B,C). Next, we added luzindole during HFSC culture to provide an in‐depth understanding of the effect of MLT on HFSCs. The results showed that after 5 days of MLT supplementation, the colony formation status of the HFSCs was significantly affected when compared with the CON and MLT + Inhib groups (Figure 6C). It was observed that after supplementation of luzindole, the colony size was not significantly affected, whereas colony formation ability was significantly decreased when compared with the MLT group (Figure 6D). These results together demonstrated that MLT enhanced the HFSC identity and promoted HFSC colony formation ability *in vitro*.

To further verify whether MLT promoted the up‐regulation of Wnt ligands in HFSCs, after 5 days of supplementation with MLT and luzindole, we collected HFSCs and evaluated the protein level of representative Wnt ligands, including Wnt5a, Wnt6 and Wnt3a (Figure 6E). The results showed that MLT significantly elevated the expression of Wnt ligands in HFSCs, further confirming our *in vivo* analysis. We next examined whether MLT supplementation could directly activate the Wnt/β‐catenin signalling pathway in DPs without HFSCs; to this end, we first isolated SOX2‐positive DP cells (Figure S6D)^51^ and subsequently established a Transwell co‐culture system with CD34‐positive HFSCs to investigate the interaction between these two cell types (Figure 6F). Notably, the protein level of β‐catenin showed no difference after MLT supplementation in DP cells, whereas when these DP cells were co‐cultured with HFSCs, the expression of β‐catenin in DP cells was significantly up‐regulated after MLT supplementation (Figure 6F). Together, these data suggested that MLT activated the Wnt/β‐catenin signalling pathway in DPs through associating with HFSC.

## 4 DISCUSSION

In the current study, we systematically investigated the effect of MLT on hair regeneration in adult mice through morphological analyses, transcriptome analyses and an *in vitro* co‐culture system. The findings revealed that MLT promoted the release of Wnt ligands from HFSCs and the secreted Wnt ligands then activated Wnt/β‐catenin signalling in DPs. The gene cascades induced by MLT exposure ultimately regulate the hair follicle cycle and promote hair regeneration. Here, we describe for the first time that MLT, which is regulated by circadian rhythms, is also capable of promoting hair regeneration in a non‐seasonally hairy mammal. This lays the factual and theoretical foundation for future research on mammalian hair growth in non‐seasonal hair‐growing animals.

In past studies, Wnt signalling has often been used as a key signalling pathway to study behaviours related to hair follicle morphogenesis.^13^, ^52^, ^53^ In the present study, we found for the first time that MLT regulated the cyclic shift of hair follicles through the Wnt signalling pathway. We found that the molecular mechanism by which MLT promoted hair follicle growth through the Wnt signalling pathway was almost identical to the results of past studies, which start with the binding of autocrine or paracrine Wnt ligands to the membrane receptor FZD; this phosphorylates downstream of GSK‐3β, and then stabilizes β‐catenin, so that it accumulates in the cytoplasm and starts the signalling cascade.^2^, ^12^ However, the stimulus for Wnt ligand release for the initiation of Wnt signalling remained ambiguous in previous studies. In our research, we found that MLT treatment significantly enhanced the expression of Wnt ligands in HFSCs, which explained the initiation of Wnt signalling in hair follicles. MLT treatment activated Wnt signalling in advance, which led to the early entry of hair follicles into the anagen phase after treatment.

Another intriguing point associated with the current study is that hair regeneration in human skin gradually decreases with the progression of aging. According to published reports, in aged mice, hair follicles exhibit prolonged telogen and shortened anagen periods,^54^ which is attributed to the altered expression of some genes associated with aging^54^; this may also lead to the accumulation of DNA damage in HFSCs, which can cause some hair follicles to permanently exit the growth cycle.^55^ Combined with our finding here that MLT promoted hair regeneration in the depilation mouse model and the fact that MLT concentrations in human serum have been demonstrated to decrease with age,^56^, ^57^ it is therefore plausible that the aging‐induced decrease in serum MLT levels may be responsible for the failure of hair regeneration in the elderly. However, many factors besides MLT concentration in serum affect human hair growth, including hair follicle stem cells, hormone level, local inflammation and subcutaneous fats.^58^ Further exploration is needed to investigate whether MLT supplementation in aged mice can abrogate aging‐induced hair regeneration failure. These studies are of significance as in‐depth research in this area may provide potential therapeutic treatments for aging‐induced hair loss. The extent of MLT in alleviating aging‐induced hair loss remains to be investigated, as this process may also involve reduced responsiveness to MLT in specific cell types in aged hair follicles. Through an in‐depth exploration of these questions, it is expected that novel insights regarding the role of MLT in hair follicle regeneration and aging‐induced hair growth failure will be identified. Although these studies on hair regeneration in mice are not directly applicable to humans, they are of significance as in‐depth research in this area may provide potential therapeutic treatments for aging‐induced hair loss as the molecular regulatory mechanisms revealed by these studies can be used as a reference for exploring how to promote human hair growth.

Our study also has some limitations. When we cultured HFSCs *in vitro*, we found that a greater number of small colonies were formed after treatment in the MLT compared with CON group, and it is not known whether this change in colony formation influenced hair folliculogenesis. Furthermore, the way in which MLT promoted the release of Wnt ligands in HFSCs remains to be explored. Moreover, given that MLT promoted HFSC colony formation, whether MLT promotes Wnt ligand secretion through enhancing the functionality of HFSCs or increasing HFSC proliferation in hair follicles *in vivo* requires further investigation.

**FIGURE 7:**
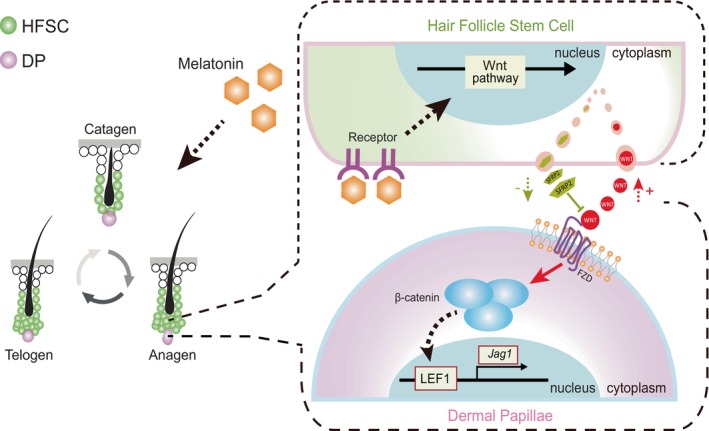
A schematic diagram illustrating how melatonin (MLT) activates the Wnt/β‐catenin signalling pathway in dermal papillae (DP) cells by promoting the release of Wnt ligands from hair follicle stem cells (HFSCs), ultimately leading to enhanced hair regeneration.

In summary, herein, we used the hair depilation mouse model to systematically clarify the role of MLT on hair regeneration and demonstrated that MLT supplementation accelerated the hair cycle. Notably, supplementation of MLT receptor antagonist luzindole significantly decreased hair regeneration in the hair depilation mouse model, indicating the critical role of MLT receptors in regulating hair follicle morphogenesis. Through comparison of transcriptome profiles between the MLT and MLT receptor antagonist luzindole‐treated groups, we determined that MLT promoted hair growth by mediating the Wnt/β‐catenin signalling pathway. Through in‐depth investigation of the mechanisms by which MLT activated the Wnt/β‐catenin signalling pathway, we found that MLT supplementation not only significantly activated Wnt/β‐catenin signalling in DPs, but also significantly up‐regulated the expression of Wnt ligands in HFSCs. Furthermore, *in vitro* incubation of MLT with HFSCs verified the up‐regulation of the protein level of Wnt ligands. Through the establishment of a DP‐HFSCs co‐culture system, we unveiled that supplementation of MLT to DPs failed to activate Wnt/β‐catenin signalling in DPs, whereas Wnt/β‐catenin signalling in DPs was activated when DP cells were cocultured with HFSCs. In summary, our results indicate that MLT activated the Wnt signalling pathway in DP cells by promoting the release of Wnt ligands from HFSCs, ultimately facilitating hair regeneration and growth (Figure 7).

## AUTHOR CONTRIBUTIONS

WG and WS conceived and designed the study. LYN and KYL performed the cell experiments. XCG and WWL performed the animal experiments. LL and HW analysed the data and revised and edited the manuscript. WG and WS supervised the project and wrote the manuscript. All authors read and approved the final version of this manuscript.

## FUNDING INFORMATION

This work was supported by the National Natural Science Foundation of China (32100683), the Natural Science Foundation of Shandong Province (ZR2021QC003), the Start‐up Fund for High‐level Talents of Qingdao Agricultural University (6651121003) and the Taishan Scholar Construction Foundation of Shandong Province (ts20190946 and tsqn202211194) of China.

## CONFLICT OF INTEREST STATEMENT

The authors declare no conflicts of interest.

## Supporting information


**Figure S1.** The body weight dynamics of mice in different groups during the treatment period. Each group comprised at least five replicates.

**Figure S2.** The effect of MLT on the expression of cell proliferation marker Ki67 and epidermal thickness. (A) Immunofluorescence staining of Ki67 in hair follicles at 20 days after depilation. Scale bars, 25 μm. (B) Statistical comparison of epidermal thickness between different groups.

**Figure S3.** Comparison of gene expression profiles between different groups. (A) Circos plots showing shared DEGs among the four groups. Shared genes are linked by purple lines. (B) Circos plots showing shared GO terms among the four groups. Shared GO terms are linked by blue lines.

**Figure S4.** Immunofluorescence staining of β‐catenin of skin tissues at 20 days after depilation. Scale bars, 50 μm.

**Figure S5.** Comparison of relevant mRNA expression levels of Wnt ligands in Successful (CON and MLT) and Failed (Inhib and MLT + Inhib) groups in day 20 skin samples, *p < 0.05, **p < 0.01, ***p < 0.001.

**Figure S6.** The effect of different concentrations of MLT on HFSCs during *in vitro* culture. (A) Bright‐field images of HFSC colonies after 1 day of supplementation with different concentrations of MLT. Scale bars, 50 μm. (B) Comparison of the HFSC marker CD34 expression in HFSCs exposed to different concentrations of MLT. Scale bars, 25 μm. (C) Comparison of colony diameter and relative CD34 intensity in HFSCs exposed to different concentrations of MLT at various time points. (D) SOX2 immunofluorescence staining of DP. Scale bars, 100 μm.

**Table S1.** The mRNA expression matrices between different groups.

**Table S2.** List of primary antibodies used in this study.

**Table S3.** List of secondary antibodies used in this study.

## Data Availability

The accession number for the RNA‐seq raw data from in this article is GSA (Genome Sequence Archive in BIG Data Center, Beijing Institute of Genomics, Chinese Academy of Sciences): CRA013709.
