# Supplementary figures and images for: Melatonin promotes hair regeneration by modulating the Wnt/β‐catenin signalling pathway
Source: Cell Prolif. 2024 May 21;57(9):e13656. doi: 10.1111/cpr.13656 (PMC11503254; doi:10.1111/cpr.13656)

Supplementary Figure 1

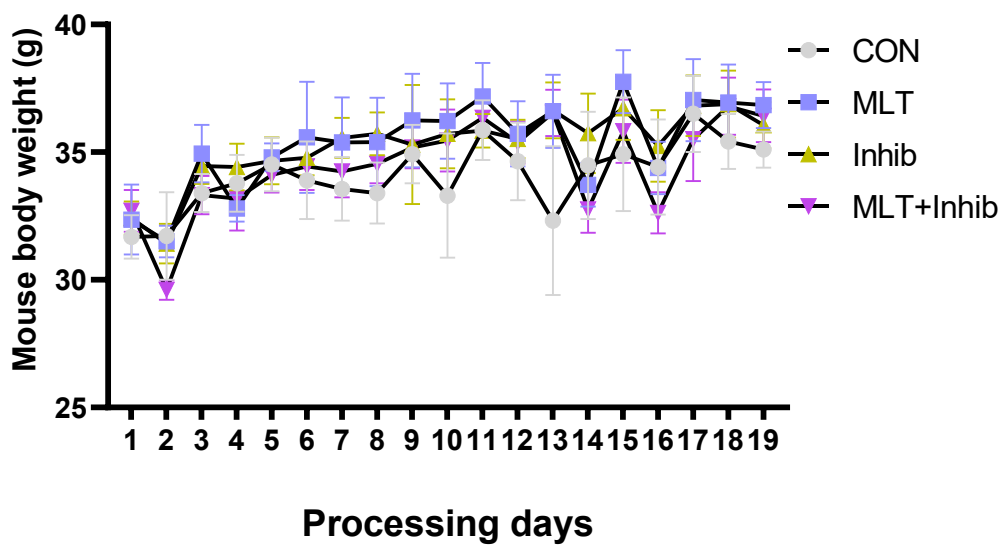

Supplement: Supplementary file 1 — Figure S1. The body weight dynamics of mice in different groups during the treatment period. Each group comprised at least five replicates. [file CPR-57-e13656-s009.pdf]

# Supplementary Figure 2

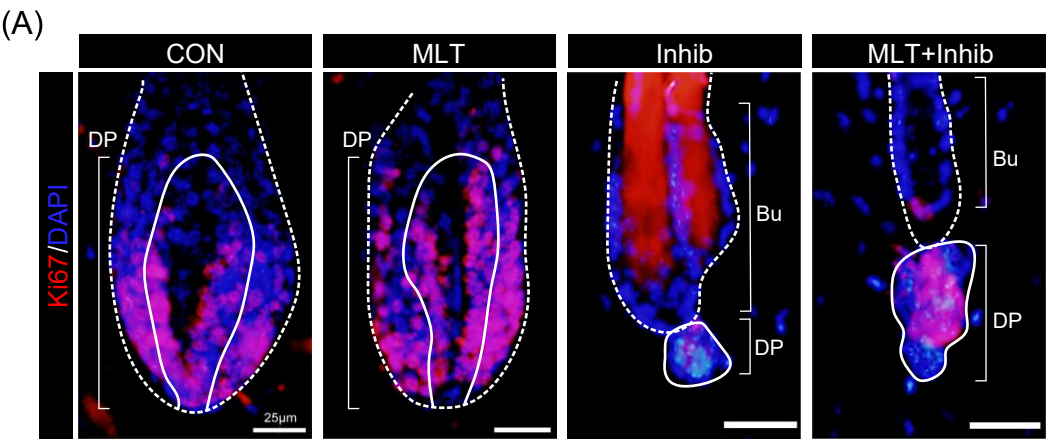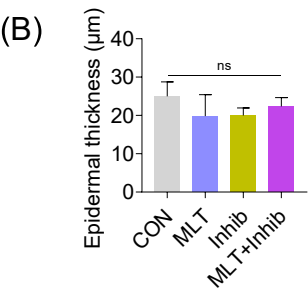

Supplement: Supplementary file 2 — Figure S2. The effect of MLT on the expression of cell proliferation marker Ki67 and epidermal thickness. (A) Immunofluorescence staining of Ki67 in hair follicles at 20 days after depilation. Scale bars, 25 μm. (B) Statistical comparison of epidermal thickness between different groups. [file CPR-57-e13656-s004.pdf]

Supplementary Figure 3

(A)

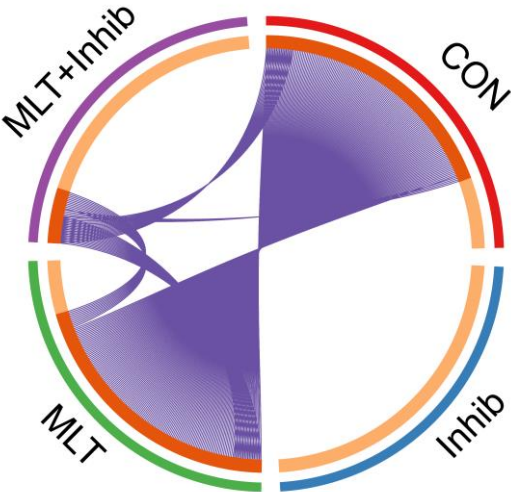

(B)

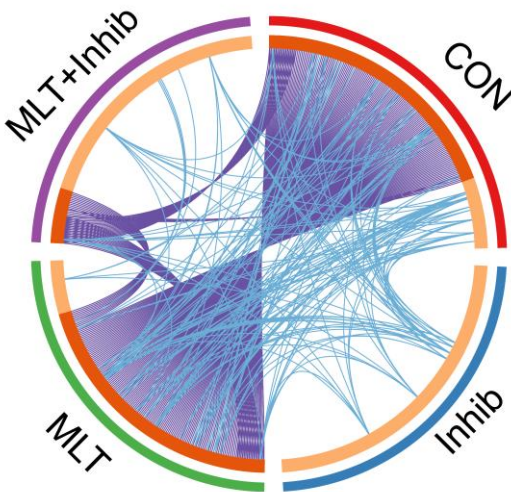

Supplement: Supplementary file 3 — Figure S3. Comparison of gene expression profiles between different groups. (A) Circos plots showing shared DEGs among the four groups. Shared genes are linked by purple lines. (B) Circos plots showing shared GO terms among the four groups. Shared GO terms are linked by blue lines. [file CPR-57-e13656-s002.pdf]

Supplementary Figure 4

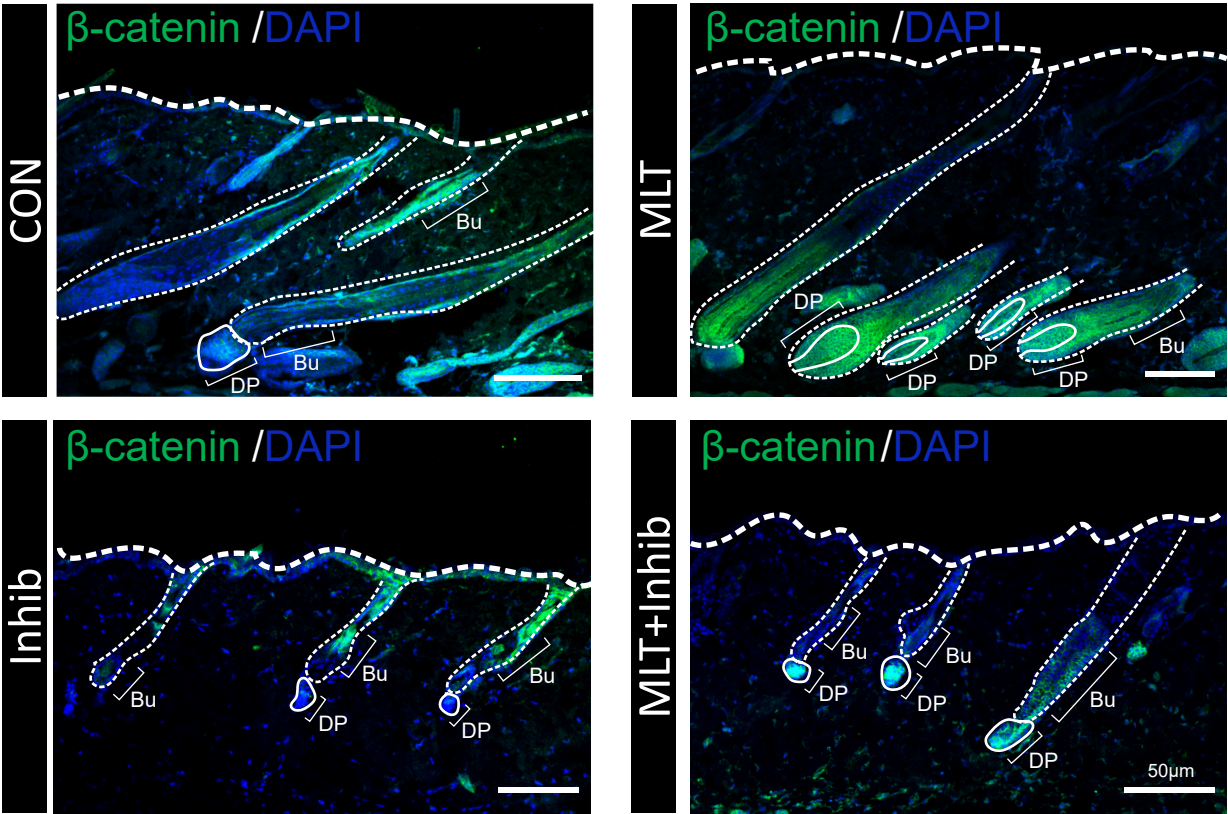

Supplement: Supplementary file 4 — Figure S4. Immunofluorescence staining of β‐catenin of skin tissues at 20 days after depilation. Scale bars, 50 μm. [file CPR-57-e13656-s001.pdf]

Supplementary Figure 5

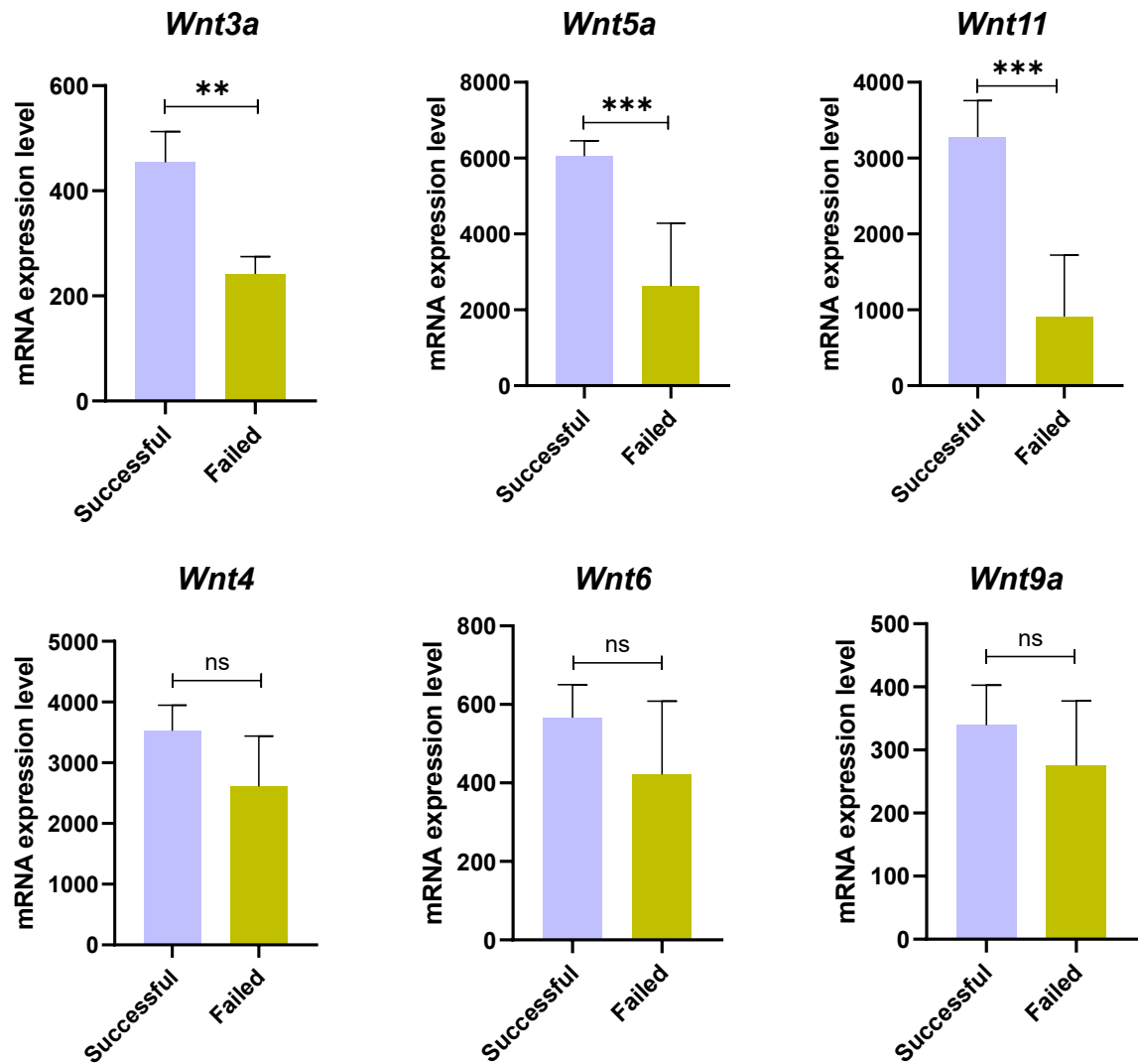

Supplement: Supplementary file 5 — Figure S5. Comparison of relevant mRNA expression levels of Wnt ligands in Successful (CON and MLT) and Failed (Inhib and MLT + Inhib) groups in day 20 skin samples, *p < 0.05, **p < 0.01, ***p < 0.001. [file CPR-57-e13656-s006.pdf]

# Supplementary Figure 6

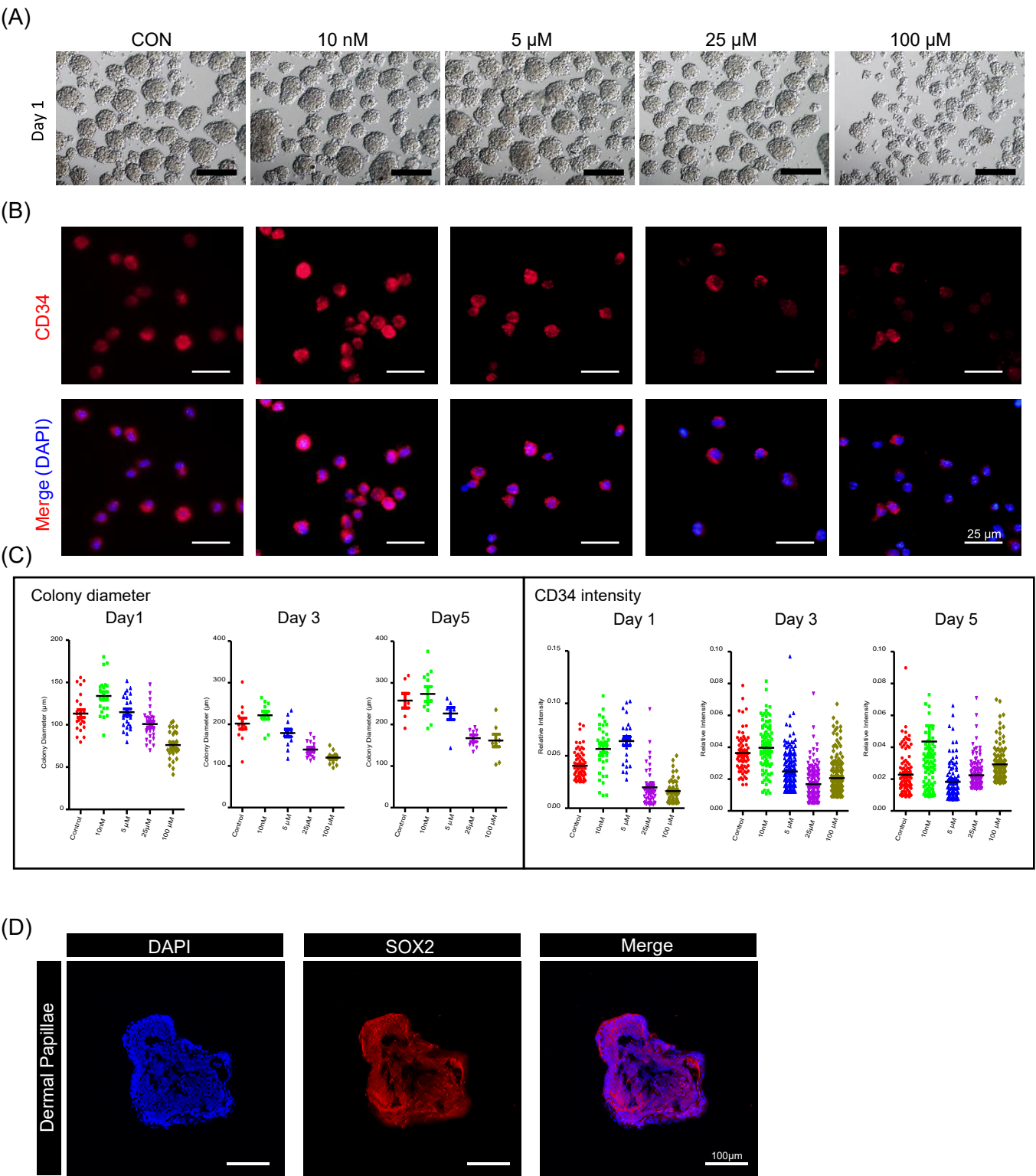

Supplement: Supplementary file 6 — Figure S6. The effect of different concentrations of MLT on HFSCs during in vitro culture. (A) Bright‐field images of HFSC colonies after 1 day of supplementation with different concentrations of MLT. Scale bars, 50 μm. (B) Comparison of the HFSC marker CD34 expression in HFSCs exposed to different concentrations of MLT. Scale bars, 25 μm. (C) Comparison of colony diameter and relative CD34 intensity in HFSCs exposed to different concentrations of MLT at various time points. (D) SOX2 immunofluorescence staining of DP. Scale bars, 100 μm. [file CPR-57-e13656-s007.pdf]
