# Supplementary material for: Melatonin promotes hair regeneration by modulating the Wnt/β‐catenin signalling pathway
Source: Cell Prolif. 2024 May 21;57(9):e13656. doi: 10.1111/cpr.13656 (PMC11503254; doi:10.1111/cpr.13656)
Supplement: Supplementary file 8 — Table S2. List of primary antibodies used in this study. [file CPR-57-e13656-s008.docx]

Supplementary Tables

Table S2. Primary antibodies Information

| Primary antibodies | Vendor | Dilution | Source |
| --- | --- | --- | --- |
| PCNA | Abcam (ab29) | 1:200 | Mouse |
| SOX9 | Abcam (ab185966) | 1:200 | Rabbit |
| KI67 | Abcam (ab15580) | 1:200 | Rabbit |
| β-Catenin | Proteintech (51067-2-AP) | 1:1000/1:200 | Rabbit |
| LEF1 | Proteintech (14972-1-AP) | 1:1000/1:200 | Rabbit |
| KRT10 | BBI Life Sciences (D260180-0025) | 1:1000 | Rabbit |
| JAGGED1 | Santa Cruz Biotechnology (sc-390177) | 1:500 | Mouse |
| KRT14 | Santa Cruz Biotechnology (sc-53253) | 1:200 | Mouse |
| WNT3A | Proteintech (26744-1-AP) | 1:1000/1:200 | Rabbit |
| WNT5A | Proteintech (55184-1-AP) | 1:1000/1:200 | Rabbit |
| CD34 | Abcam (ab81286) | 1:200 | Rabbit |
| CD34 | Santa Cruz Biotechnology (sc-74499) | 1:500 | Mouse |
| SFRP2 | Santa Cruz Biotechnology (sc-365524) | 1:500 | Mouse |
| KRT15 | Proteintech (10137-1-AP) | 1:200 | Rabbit |
| WNT6 | Proteintech (24201-1-AP) | 1:1000 | Rabbit |
| GAPDH | Affnity (Af7021) | 1:1000 | Rabbit |
| P-β-catenin  SOX2 | HuaBio (JE59-59)  Abcam (Ab92494) | 1:1000  1:200 | Rabbit  Rabbit |
