# Supplementary material for: Melatonin promotes hair regeneration by modulating the Wnt/β‐catenin signalling pathway
Source: Cell Prolif. 2024 May 21;57(9):e13656. doi: 10.1111/cpr.13656 (PMC11503254; doi:10.1111/cpr.13656)
Supplement: Supplementary file 9 — Table S3. List of secondary antibodies used in this study. [file CPR-57-e13656-s003.docx]

Table S3. Secondary antibodies Information

| Secondary antibodies | Vendor | Dilution |
| --- | --- | --- |
| Goat Anti-Rabbit IgG H&L (Alexa Fluor® 488) | Abcam (ab150077) | 1:200 |
| Donkey Anti-Rabbit IgG H&L (Alexa Fluor® 555) | Abcam (ab150074) | 1:200 |
| Donkey Anti-Mouse IgG H&L (Alexa Fluor® 555) | Abcam (ab150106) | 1:200 |
| Goat Anti-Mouse IgG H&L (Alexa Fluor® 488) | Abcam (ab150113) | 1:200 |
| HRP-conjugated goat anti-Mouse IgG | Beyotime (A0216) | 1:1000 |
| HRP-conjugated goat anti-rabbit IgG | Beyotime (A0258) | 1:1000 |
